# Immune-Based Anti-Staphylococcal Therapeutic Approaches

**DOI:** 10.3390/microorganisms9020328

**Published:** 2021-02-06

**Authors:** Bonggoo Park, George Y. Liu

**Affiliations:** 1 Cedars Sinai Medical Center, Division of Pediatric Infectious Diseases and the Immunobiology Research Institute, Los Angeles, CA 90048, USA; parkbg35@gmail.com; 2 Department of Pediatrics, University of California San Diego, La Jolla, CA 92093, USA

**Keywords:** methicillin-resistant *S. aureus* (MRSA), immune boosting strategy, innate defense regulator peptide (IDR-1), cyclic-di–guanosine monophosphate (c-di-GMP), chronic granulomatous disease (CGD), neutrophil extracellular traps (NETs), Hypoxia-inducible factor (HIF)-1α, CCAAT/enhancer binding protein (C/EBPε), mesenchymal stem cells (MSC), serum therapy

## Abstract

Widespread methicillin-resistant *Staphylococcus aureus* (*S. aureus*) infections within community and healthcare settings are responsible for accelerated development of antibiotic resistance. As the antibiotic pipeline began drying up, alternative strategies were sought for future treatment of *S. aureus* infections. Here, we review immune-based anti-staphylococcal strategies that, unlike conventional antibiotics, target non-essential gene products elaborated by the pathogen. These strategies stimulate narrow or broad host immune mechanisms that are critical for anti-staphylococcal defenses. Alternative approaches aim to disrupt bacterial virulence mechanisms that enhance pathogen survival or induce immunopathology. Although immune-based therapeutics are unlikely to replace antibiotics in patient treatment in the near term, they have the potential to significantly improve upon the performance of antibiotics for treatment of invasive staphylococcal diseases.

## 1. Introduction

Infectious diseases are a leading cause of deaths worldwide especially in economically disadvantaged countries [1]. High infection burden has propelled a greater reliance on antibiotics, which over time has led to significant antibiotic resistance development in most human pathogens, to the extent that antibiotic development has not and will not be able to keep up [2]. As antibiotics became more limiting, especially for treatment of Gram-negative pathogens, investigators began to seek alternative ways to treat infections. The *S. aureus* field was a prime example of a hub where, driven by need, investigators have gone to great length to develop new ways to fight a pathogen that have been a major threat to society. *S. aureus* is a pathogen with a large armamentarium of virulence factors that has enabled it to induce a wide range of infections [3,4]. Resistance of *S. aureus* to penicillin emerged shortly after penicillin was first introduced [5], and with frequent antibiotic use, *S. aureus* resistant to penicillin and methicillin became abundant in healthcare settings in the 1980s. In the past two decades, clones of community-associated methicillin-resistant *S. aureus* (CA-MRSA) emerged outside of hospitals in individuals with no risk factors. Unexpectedly, the CA-MRSA strains were demographically, clinically, and microbiologically distinct, from their hospital-associated counterpart [6,7,8]. At the height of the epidemic, the CA-MRSA strains accounted for close to fifty percent of all soft tissues staphylococcal infections in the United States [6]. In association with increased use of antibiotics to treat MRSA infections, antibiotics that were once designated as last line antibiotics such as vancomycin were noted to develop reduced efficacy [9]. For example, in what is referred to as glycopeptide creep, *S. aureus* strains with increased vancomycin resistance were reported more frequently and resistance was correlated with poorer clinical outcomes [10,11,12]. Although a few other classes of antibiotics are currently available to treat MRSA infections, emergence of resistance in *S. aureus* can already be found to the newest antibiotics developed in the past years [13,14]. It is well acknowledged that, long term, antibiotics cannot be the solution to combating major pathogens such as *S. aureus*. As alternative strategies, investigators have developed an array of non-antibiotic approaches that act on the pathogens to weaken the microbe, or the host to boost immune defenses. Other approaches aimed to boost existing microbiota that compete against *S. aureus* for colonization [15,16]. Below, we will only discuss immune-based strategies that target either host and microbial factors and refer the reader to many reviews on related topics [3,4,17].

## 2. Immune Boosting Strategies

Immune boosting strategies that enhance clearance of pathogens have been advocated by the National Research Council as a non-conventional way to combat antimicrobial resistance [18]. Unlike strategies directed at microbial virulence factors that target individual species of microbes, immune stimulation approaches target antimicrobial pathways within the immune system that are particularly effective against certain pathogen classes. Although immune boosting agents are unlikely to rival antibiotics in term of antimicrobial efficacy, they can synergize with antibiotics to optimize the outcome of hard-to-treat infections. However, immune-based strategies pose a number of issues that could make them unsuitable for clinical use: Since they act on the host immune system, their efficacy is highly dependent on the state of the host’s immune system. For example, chemokine therapy would find no application if no neutrophils are available for recruitment in patients on chemotherapy. Induction of excessive inflammation could lead to immunopathology that is worse than the infection. As with exposure to any reagents that threaten the survival of the pathogen, pathogens could develop resistance to the reagent and the immune pathway and, in effect, induce a state of “immunocompromise” of the host to that pathogen. Understandably, the constraints posed by these and additional issues such as cost, favorable pharmacokinetics, and stability further limit the number of agents that find suitable indications in the clinics.

### 2.1. Targeting Neutrophils and Related Pathways

Various immune strategies that aim to limit staphylococcal infections have sought to stimulate neutrophils or neutrophil-related antimicrobial factors. Congenital neutrophil deficiencies such as chronic granulomatous diseases, severe congenital neutropenia and specific granule deficiency, which present with severe staphylococcal infections early during childhood, have made evident the clinical importance of neutrophils in staphylococcal infections [19,20] and suggested that control of *S. aureus* infections could be enhanced by targeting neutrophils.

Neutrophils are the first immune cells to mobilize from the bloodstream to the site of infection after localized *S. aureus* infection [21]. Recruitment of neutrophils from the bone marrow and the vasculature occurs through chemokines released by *S. aureus*, local parenchymal cells, and myeloid cells. Following recruitment, neutrophils effectively unleash prepacked granules containing highly microbicidal peptides, proteases, reactive oxygen species (ROS), and reactive nitrogen species (RNS) upon the phagocytosed *S. aureus*. Neutrophil extracellular traps (NETs) secreted by dying neutrophils also limit the systemic spread of *S. aureus*. Strategies that boost selective steps in neutrophil functions have shown promise as anti-staphylococcal therapeutics.

#### 2.1.1. Neutrophil Store

Neutrophils that reside within the bone marrow or the vasculature are first responders to bacterial infections. Although the neutrophil number increases with the severity of staphylococcal infection, in the setting of immunocompromise such as chemotherapy or infant prematurity, the host has reduced capacity to generate new neutrophils. Granulocyte colony-stimulating factor (G-CSF) and Granulocyte-Macrophage colony stimulating factors (GM-CSF) are established therapeutics that promote the recovery of neutrophil and monocyte cell counts in neutropenic cancer patients undergoing chemotherapy particularly in the setting of infections [22]. Recovery of neutrophils in those settings is required for effective clearance of pathogens such of *S. aureus*, and as a standard protocol, antibiotics are continued indefinitely in infected patients until neutrophil count is sufficiently recovered [23].

An alternative approach to G-CSF as treatment for infections in the neutropenic host is the transfusion of freshly harvested neutrophils [24]. This approach has been adopted infrequently as a last line measure against severe fungal infections where mortality is particularly high, but has also been reported in association with treatment of severe *S. aureus* infections in neutropenic hosts [25]. The approach is labor and resource intensive since neutrophil half-life is short and poses many technical challenges. Overall, meta-analysis has not shown a clear benefit associated with the treatment [25], although a more promising approach has been described that uses a preactivated neutrophil cell line to clear candida infection. Pre-activation and prior irradiation of the myeloid cell line HL60 led to non-proliferation of the infused cells and improved survival of the mice infected systemically with candida with minimal noted toxicity [26].

#### 2.1.2. Myeloid Cell Recruitment

Neutrophil recruitment to the infected tissues requires the local release of chemokines into the circulation by resident and myeloid cells. *S. aureus*, additionally, secretes a chemotactic formylated tripeptide N-formylmethionyl-leucyl-phenylalanine (fMLP) that further recruits neutrophils to the infection site [27]. However, the pathogen also secretes severe proteins that interfere with neutrophil recruitment, including the Chemotaxis Inhibitory Protein (CHIP) and the Extracellular adherence protein (Eap), which, respectively, block neutrophil recognition of chemotactic factors [28] and neutrophil binding to endothelial adhesion molecule, intercellular adhesion molecule-1 (ICAM-1) [29] required for neutrophil adhesion, diapedesis, and extravasation from the bloodstream to the site of infection. Administration of chemokines that overwhelm these staphylococcal strategies could clearly improve clearance of *S. aureus*.

**Figure 1:**
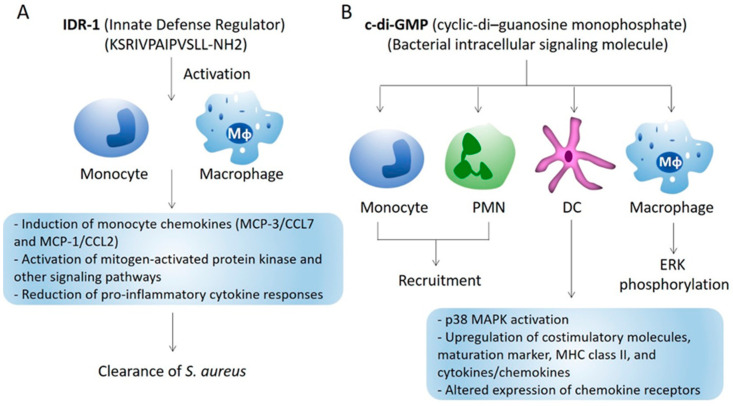
Immune-based strategies targeting chemokines. (**A**) IDR-1, a synthetic host defense peptide derivative, activates immune signaling pathways that increase the levels of infection clearing chemokines, while suppressing pro-inflammatory cytokines such as tumor necrosis factor-α (TNF-α), leading to S. aureus clearance with little undesirable toxicities. (**B**) A potent bacterial immunostimulatory molecule C-di-GMP induces monocyte/granulocyte recruitment, increased expression of costimulatory molecules, maturation marker, MHC class II, cytokines/chemokines and altered expression of chemokine receptors. The peptide also induces p38 mitogen-activated protein kinase (MAPK) activation in DCs and extracellular signal regulated kinase (ERK) phosphorylation in macrophages.

The innate defense–regulator peptide-1 (IDR-1) is an immunomodulatory peptide with chemotactic activity for neutrophils, monocytes and macrophages [30,31]. IDR-1 is not directly antimicrobial and induces myeloid cell recruitment through mitogen-activated protein kinase and other pathways. IDR-1 and synthetic immune defense regulator peptides offer the therapeutic benefit that they have immune modulator activities and are not associated with toxicities [30,31]. Anti-staphylococcal benefit of IDR-1 is demonstrated by protection against MRSA infection following local or systemic administration of IDR-1 [30]. When incubated with human neutrophils, IDR-1 acted as a partial agonist for the formyl peptide receptor and induced chemotactic migration of the neutrophils without producing the superoxide anion and intracellular calcium that are associated with formyl peptide stimulation [31]. Because of their ease of manufacturing, innate defense regulator peptides and their analogs are actively studied for their potential to serve as human therapeutics (Figure 1A).

Another chemotactic compound, cyclic dinucleotide, cyclic-di–guanosine monophosphate (c-di-GMP), is a microbial molecule sensed in the cytosol of mouse and human cells via the stimulator of interferon genes (STING) immunosurveillance pathway [32]. C-di-GMP serves as a second messenger with intracellular signaling function in many species of bacteria but is not made in eukaryotic cells [32]. When injected intraperitoneally into mice, it induces neutrophil and monocyte recruitment to the site of injection. The peptide can further increase expression of costimulatory molecules, maturation marker, major histocompatibility complex (MHC) class II, and cytokines and chemokines, and altered expression of chemokine receptors in human immature dendritic cells (DC), leading to enhanced T cell stimulatory activity [30]. Intramammary application of c-di-GMP has been shown to ameliorate *S. aureus* infection in a mouse model of mammary infection [33] (Figure 1B).

### 2.2. Targeting Specific Neutrophil Functions

#### 2.2.1. ROS and RNS

One of the best characterized genetic diseases that underline the importance of neutrophils in *S. aureus* defense is chronic granulomatous disease (CGD), a condition characterized by recurrent infections with bacteria and fungi as well as inflammatory complications [34]. In patients with CGD, phagocytes fail to produce superoxide and its oxygen metabolites, which can be traced to defects in the NADPH oxidase complex. CGD exhibits heterogeneity of inheritance with the majority of cases shown to be X-linked and a minority, autosomal recessive [35]. Interferon-γ (IFN-γ) has been used for decades as a routine prophylactic agent in CGD patients that reduces the incidence of infections [34]. IFN-γ increased superoxide generation in neutrophils and macrophages from patients with the gp91phox X-linked CGD, but not from myeloid cells from patients with “classic” CGD (that showed no detectable baseline superoxide generation) or autosomal variant CGD [34,35]. IFN-γ treatment of CGD patients is associated with increased neutrophil production of nitric oxide (NO) and bactericidal capacity when activated by fMLP [36]. Although IFN-γ amelioration of neutrophil activity is likely responsible for the reduced incidence of infections in CGD patients, it is unclear if the benefit of IFN-γ therapy comes primarily from its influence on ROS or RNS production.

#### 2.2.2. Antimicrobial Peptides

Antimicrobial peptides are a diverse class of innate immune molecules with antibacterial, antiviral, or antifungal activity [37]. They are secreted by neutrophils and epithelial cells from the skin and mucosal surfaces during *S. aureus* infections. Natural or analogs of antimicrobial peptides are intensely studied because of their potential to provide direct, rapid, and potent broad spectrum killing of bacterial pathogens as an alternative to traditional antibiotics [38]. Some natural cationic antimicrobial peptides with immunomodulatory function improve infection outcome in spite of having no direct antimicrobial activity [38,39]. Although antimicrobial peptides hold significant promise for treatment of infections, their stability, half-life, potential for toxicity, and induction of resistance to endogenous antimicrobial peptides, as well as manufacturing issues remain barriers to their clinical application [37,38].

**Figure 2:**
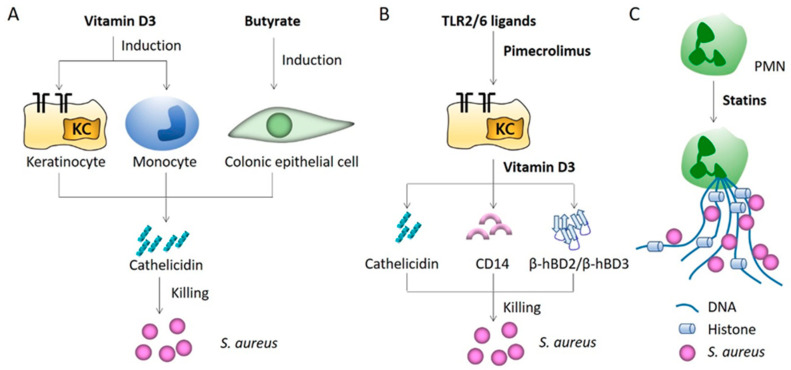
Immune-based strategies antimicrobial peptides and NETs. (**A**) Vitamin D3 induces cathelicidin expression in keratinocytes and monocytes, whereas butyrate induces cathelicidin in colonic epithelia, leading to the enhanced antimicrobial activity against S. aureus. (**B**) Calcineurin inhibitor pimecrolimus enhances expression of cathelicidin, CD14, and human β-defensin-2 and β-defensin-3 in response to TLR2/6 ligands in keratinocytes and leads to inhibition of S. aureus growth. Pimocrolimus function can be enhanced by 1,25(OH)2 vitamin D3. (**C**) Statins enhance S. aureus clearance using antibacterial DNA-based extracellular traps in human and murine myeloid cells, by targeting the sterol biosynthesis pathway. KC: keratinocytes-derived chemokine; PMN: polymorphonuclear neutrophil.

An indirect approach to harnessing antimicrobial peptides is through the use of vitamin D3 and butyrate, which, respectively, induce cathelicidin expression in keratinocytes and monocytes (vitamin D3) and in colonic epithelial cells (butyrate) [40]. During the course of *S. aureus* skin infection, keratinocytes rely on toll-like receptors (TLR) and antimicrobial peptides to appropriately recognize and respond to injury or microbes [41]. Upon surrounding the wound, keratinocytes increase expression of microbial pattern recognition receptors CD14 and TLR2 and cathelicidin to limit the infection [42]. 1,25(OH)2 vitamin D3 simulates these injury responses by enabling keratinocytes to recognize microbial components through TLR2 and respond by cathelicidin production [42] (Figure 2A). In a recent study, Buchau and colleagues demonstrated that another agent, pimecrolimus, a calcineurin inhibitor, enhanced expression of cathelicidin, human β-defensin-2, and β-defensin-3 in response to TLR2/6 ligands in keratinocytes. Some of the responses are further augmented by 1,25(OH)2 vitamin D3 and lead to growth inhibition of *S. aureus* [41] (Figure 2B). Consistent with the role of vitamin D in the induction of antimicrobial peptides in human skin, individuals with low serum vitamin D levels had a statistically significant increased risk of MRSA carriage in a secondary data analysis of the National Health and Nutrition Examination Survey 2001–2004 for the non-institutionalized population of the USA [43].

#### 2.2.3. NETS

Neutrophil extracellular traps (NETs) are a programmed defense system deployed by dying neutrophils to control and localize infections [44]. NETs are composed of chromatin with bound granule proteins that together entrap and kill both Gram-positive and Gram-negative pathogens and neutralize their virulence factors to ensure they do not spread systemically [45]. Statin, an inhibitor of 3-hydroxy 3-methylglutaryl coenzyme A (HMG-CoA) reductase in cholesterol biosynthesis, shows NETs enhancing activity [46] when incubated with human and murine neutrophils (Figure 2C). Generation of NETs is shown to be dependent on intermediates of the sterol synthesis pathway. Blockade of HMG-CoA by statins or siRNA inhibits the sterol synthesis pathway and generation of the extracellular traps and thereby improved myeloid cell clearance of *S. aureus* [46]. The antimicrobial effect of statins is corroborated by the improved infection outcome associated with statin therapy in humans and mice [46].

### 2.3. Targeting Regulators of Neutrophils

Engagement of regulatory pathways instead of specific effector molecules permits a broader and potentially more effective assault on *S. aureus* but also risks the induction of more significant bystander immunopathology. Only two strategies have successfully controlled staphylococcal infections without inducing significant toxicities in pre-clinical infection models [47,48].

**Figure 3:**
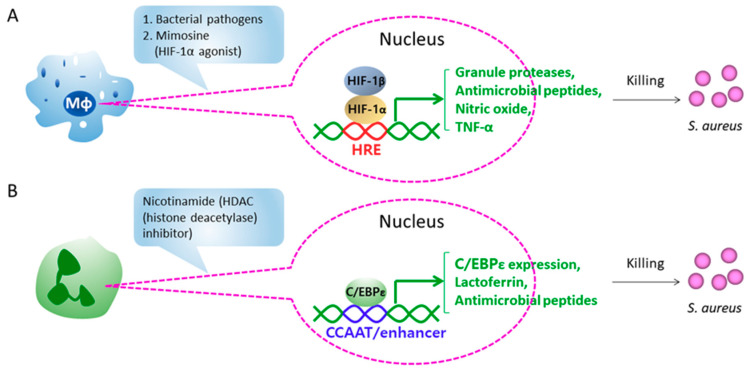
Immune-based strategies targeting regulators. (**A**) HIF-1α is activated on exposure to bacterial pathogens and induces the production of granule proteases, antimicrobial peptides, nitric oxide, and TNF-α in host phagocytes, suggesting HIF-1α as a therapeutic target for enhancing host defense. HIF-1α agonist mimosine pharmacologically augments antimicrobial capacity of human phagocytes and whole blood to kill S. aureus. (**B**) HDAC inhibitor nicotinamide enhances mRNA and protein levels of C/EBPε and several downstream effector molecules such as antimicrobial peptides and proteins in neutrophils.

Hypoxia-inducible factor-1α (HIF-1α), as the master regulator of cellular immune responses to hypoxia, has been an attractive target of antimicrobial therapy [49]. HIF-1α is induced within the hypoxic microenvironment established by infection and leads to the production of granule proteases, antimicrobial peptides, nitric oxide, and TNF-α within phagocytes [47,50]. In studies using mice lacking HIF-1α strictly in the myeloid cell lineage, bacterial infection elicited lower bactericidal activity and increased systemic spread from the initial tissue focus in the mutant mice [50]. Application of the HIF-1α agonist mimosine augmented dose-dependent killing of *S. aureus* by human phagocytes and whole blood while reducing the lesion size in a murine skin infection model [47] (Figure 3A).

We have previously described another immune boosting strategy that was inspired from studies of the neutrophil specific granule deficiency condition (SGD), a rare congenital immunodeficiency condition that is marked by enhance susceptibility to *S. aureus* and *P. aeruginosa* infections [19]. SGD neutrophils exhibit atypical bilobed nuclei, lack expression of at least one primary and all secondary and tertiary granule proteins, and possess defects in chemotaxis, disaggregation, receptor upregulation, and bactericidal activity, which contribute primarily to the immune deficiency [19]. The functional loss of the myeloid transcription factor CCAAT/enhancer binding protein ε (C/EBPε or CEBPE) was shown to be responsible for the development of SGD in several patients [19]. The murine SGD model subsequently generated by deletion of *CEBPE* demonstrated defects in neutrophil and eosinophil granule gene expression and abnormalities in macrophage maturation and function, consistent with C/EBPε regulating essential innate immune functions [19,51,52]. We showed that C/EPBε^−/−^ mice were exquisitely susceptible to *S. aureus* infections as evidenced by failure to clear *S. aureus* and, in fact, growth of *S. aureus* within neutrophils. Consistent with these findings, depletion of neutrophils in these mice led to improved staphylococcal infection outcome [48]. Histone deacetylase (HDAC) inhibitor nicotinamide enhanced CEBPE expression, increased expression of several downstream effector molecules including lactoferrin and cathelicidin antimicrobial peptide, and enhanced microbicidal activity of wild-type murine and human neutrophils (Figure 3B). When applied at high dose (300 mg/kg/d), nicotinamide improved the clearance of MRSA in wild-type mice [48]. Nicotinamide treatment was also associated with reduced systemic IL-2 and IFN-γ secretions and improved survival of mice after a lethal staphylococcal enterotoxin B challenge [53].

### 2.4. Beyond Neutrophils

#### 2.4.1. Cell Transfusion with Platelets, Macrophages, MSCs

More recent studies have highlighted a key role of macrophages in control of *S. aureus* through orchestration of defenses as well as contributing to direct antimicrobial activities [54,55]. In studies utilizing macrophages as cell therapy for antibiotic-resistant bacterial infections, monocyte-derived macrophages which can be generated, harvested, and cryopreserved effectively killed *S. aureus*, as well as Gram-negative pathogens in vitro and protected against experimental lethal peritonitis and lung infection [54].

Platelet-based therapy has also demonstrated potential utility to combat staphylococcal infections based on the range of antimicrobial proteins and peptides contained in their granules with demonstrated broad-spectrum antimicrobial and chemotactic activities [56]. In pre-clinical studies, application of platelet-rich plasma therapy improved outcome of staphylococcal wound and bone infections through its antimicrobial activities [57,58]. Although platelet-rich plasma is not as effective as antibiotic treatment, it displays synergy with antibiotics and has demonstrated efficacy in many clinical trials, including treatment of patients with diabetes foot ulceration in a prospective randomized trial [59,60].

Exploration of human stem cells has also shown the potential of these cells as anti-staphylococcal therapeutics. Studies have showed that the adipose-derived human mesenchymal stem cells (MSC) significantly inhibited the growth of *S. aureus* under standard culture conditions with or without the continued presence of adipose stem cells (ASCs) through MSC production of the cationic antimicrobial peptide, LL-37 [61]. In addition, treatment of ASCs with 1,25(OH)2 vitamin D3 elevated LL-37 expression and enhanced their antimicrobial activity, whereas a vitamin D receptor inhibitor, GW0742, blocked the antimicrobial activity of MSCs [61]. When introduced into rats, bone marrow-derived MSCs enhanced bacterial clearance, suppressed the expression of cytokines and chemokines and promoted healing of the wound, in comparison to the fibroblast control groups [62]. In dairy cows infected to produce staphylococcal mastitis, allogenic bovine fetal MSC injected into the mammary glands [63] reduced bacterial count in the milk without inducing adverse clinical effects or activation of inflammatory responses in peripheral blood lymphocytes [63].

#### 2.4.2. Training and Reprograming the Innate Immune System

Trained immunity is a recent and widely studied phenomena with significant implication for therapeutics, as well as pathogenesis of human diseases [64]. In trained immunity, various types of immune cells, including macrophages and monocytes, undergo metabolic and epigenetic changes after exposure to pathogen-associated molecular patterns (PAMPs) such as nucleotide-binding oligomerization domain-containing protein 2 (NOD2) and β-glucan that activate specific immune pathways [64]. The induced changes can persist for months to years and can protect the host from bacterial, viral, or fungal infections. Ligands that induce innate immune training have been investigated in *S. aureus* models of orthopedic, as well as systemic infections. Pretreatment with zymosan, a particulate form of β-glucan, was associated with mild transient temperature elevation but enhanced the host’s clearance of *S. aureus* and decreased systemic pro-inflammatory cytokine levels when applied up to 8 weeks innate training [65,66]. Immune training drugs would be beneficial to patients with frequent admission to hospitals because of infections secondary to underlying anatomic, surgical, or immune issues.

Another iteration of immune reprograming in skin and abscesses consists of a switch of macrophage phenotype from M1 to M2 [67]. Neutrophils and M1 proinflammatory responses are normally needed for control *S. aureus* infection during the initial stage of infection [68]. However once established, continued proinflammatory immune response becomes futile and a switch or reprograming of the immune system towards M2-like macrophages drives the eventual clearance of *S. aureus* per one report [67]. Reprograming appears to occur through peroxisome proliferator-activated receptor-γ (PPAR-γ) which reduces the oxygen and glucose level in the abscess and increases antimicrobial peptides. Application of rosiglitazone, a PPAR-y agonist, led to a reprogrammed innate immune response and clearance of *S. aureus* [67].

### 2.5. Targeting Microbial Components with Antibody Therapeutics

Immunization is one of the oldest and arguably the most effective immune-based therapy developed by mankind. Immunization, both active and passive, can effectively prevent pathogens from inducing disease by blocking the pathogen from taking a foothold in tissues, surviving within the host environment, or causing tissue damages. Vaccination, when successfully executed, can wipe out diseases, reduce antibiotic use, and directly address antibiotic resistance issues. Thus, development of an effective human staphylococcal vaccine has been the holy Grail of the *S. aureus* field for the past decades, although preclinical vaccine studies date much further back [69]. Here we focus only on discussions of the general direction the staphylococcal field has taken on antibody-based therapy and will refer the readers to the many excellent published reviews [69,70,71].

#### 2.5.1. Serum Therapy

Serum therapy has been successfully used early in the 20th century against various infectious diseases such as pneumococcal invasive diseases [72]. Its use was abandoned because of immunologic complications and more especially hypersensitivity due to heterologous sera. Intravenous immunoglobulin (IVIG), a concentrated product of the serum, has been used for treatment of severe infections primarily to neutralize toxins elaborated by staphylococcal strains. Although a meta-analysis showed a benefit of IVIG therapy for improving mortality, inclusion of only high-quality trials indicated no clear benefit for IVIG [58]. More recently, advances in humanized monoclonal antibody technology have lowered complication rates in association with antibody therapy and have led to a more rapid introduction of immunotherapy into clinical settings, including trials of monoclonal antibodies targeting staphylococcal antigen targets [73]. Generally, staphylococcal passive and active immunizations that have been brought to clinical trials could be broadly divided as targeting cell surface molecules to promote opsonophagocytosis, or toxins to block toxin-induced immunopathology.

#### 2.5.2. Enhancing Opsonophagocytosis

Immunization strategies that seek to enhance opsonophagocytic killing of pathogens have been most successful with microbes encoding a prominent capsule. Thus, childhood vaccines that target *H. influenzae* and *S. pneumoniae* capsule have dramatically reduced invasive diseases caused by these major pathogens [74,75]. *S. aureus*, in comparison, makes a small capsule and relies on a broader array of virulence factors to establish infection in different tissues. Targeting the capsular polysaccharide 5 and 8 through active immunization in an early human trial did not confer robust protection [76,77]. Therefore, it became clear that developing a successful vaccine that protects against all staphylococcal diseases would be difficult. A landmark paper suggested the potential promise of targeting a group of staphylococcal surface determinants that are highly conserved across *S. aureus* strains [78]. These include Clumping factor A and B (ClfA and ClfB), Iron-regulated surface determinant A and B (IsdA and IsdB), Capsular Polysaccharide 5 and 8 (CP5 and CP8), Serine–aspartate repeat protein D and E (SdrD and SdrE), and Manganese binding protein C (MntC) [79]. Despite robust preclinical immunization data on vaccinating against these antigens, passive or active vaccines that targeted these antigens singly or in combination were unsuccessful in clinical trials. Apart from a study of polyclonal antibodies against CP5 and CP8 which effectively shortened the length of stay for patients with *S. aureus* bacteremia but not in preventing invasive infection in neonates [80,81], trials that targeted iron surface determinant B (IsdB), manganese transport protein C (MntC), and CP5/8 offered no protection to date [69].

#### 2.5.3. Neutralization of Toxins

The other major vaccine strategy to target staphylococcal toxins is grounded on human data showing prominent roles of toxins in certain staphylococcal diseases. Historically, patients who succumb to staphylococcal toxic shock syndrome have low antibody to toxic shock syndrome toxin (TSST) [82,83]. Anti-α-toxin antibody development after staphylococcal infections correlated with protection from recurrence of infection within 12 months [84]. Therefore, it has been argued that, while toxin neutralization would not be expected to fully prevent occurrence of *S. aureus* infections, clinical evidence of toxin involvement in human diseases provides a more compelling argument for targeting toxins in vaccine trials [69].

Among toxins, α-toxin has been the major focus of many vaccine trials because of the abundance of literatures pointing to its important roles in various staphylococcal diseases, especially lung and skin infections [85]. Animal models and experimental data suggest that α-toxin engage surface receptors of sensitive immune host cells including a disintegrin and metalloproteinase domain-containing protein 10 (ADAM-10), attaching to the cell surface and perforating it, and thus disabling the cells [86,87]. At epithelial barriers, sublytic α-toxin activation of ADAM10 protease leads to cleavage of junction protein E cadherin which leads to disruption of barrier [86]. To date, monoclonal antibodies against α-toxin or several related toxins based on shared epitopes (α-toxin, PVL, LukED, γ-hemolysin AB and CB (HlgAB, HlgCB)) have been developed and tested in multiple human trials. Both trials failed to reach their primary endpoint for reducing the occurrence of staphylococcal pneumonia in mechanically ventilated patients [69,88,89].

It remains unclear why all staphylococcal vaccines taken to clinical trials have failed despite adjusted strategies to target multiple and different types of antigens. Alternative approaches that focus on T cell rather than humoral responses, as has been advocated by many groups, have not yet been fully explored [90,91]. Importantly, addressing fundamental differences in humans and mice that have yielded vastly different staphylococcal vaccine outcomes should be a priority and the focus for the field for the years to come.

## 3. Conclusions

While non-antibiotic approaches are needed to address the potential antibiotic shortage in the coming decades, it could be argued that alternative immune-based approaches are needed to address more immediate needs. Front and center, the lack of an effective vaccine has allowed *S. aureus* infections to remain rampant at the expense of expanding antibiotic resistance. This clearly is the priority of the field to stem the tide of antibiotics resistance. Yet, with every active and passive immunization trials having failed, simple tweaks of the target antigens are unlikely to bring about success. The field must now reexamine why the vaccines failed and perhaps hold off on further trials until a reasonable explanation is proposed for the failed human trials.

For treatment of active *S. aureus* infection, antibiotics remain the gold standard against which all novel therapeutics are compared. As most immune-based therapeutics are unlikely to rival antibiotics in term of cost and efficacy, their acceptance as a replacement for antibiotics is not likely. However, far from being unimportant, immune-based therapies are needed to improve upon the sub-par performance of antibiotics in a variety of infectious conditions. For example, even with appropriate antibiotic treatment, mortality associated with staphylococcal bacteremia can be unacceptably high at 40 to 50 percent [92]. Infections that are confined to heart valves and bones require months of treatment to eradicate the pathogen [93] at the cost of increasing antibiotic resistance. In the absence of a working immune system, as is the case of neutropenic oncologic patients, treatment of staphylococcal invasive diseases is prolonged and prone to relapse until neutrophil count is recovered [23]. In the latter case, G-CSF has proven to be an ideal adjunct therapy that dramatically enhances efficacy of conventional antibiotics by hastening the recovery of neutrophil count [22].

Most published studies that advocate for the use of novel immune-based therapeutics have failed to take into account real-life clinical settings. Immune-based therapies are usually tested on young adult laboratory animals that nowhere mimic the patients with comorbid conditions who would be most in need of such therapeutics; in the setting of immunocompromise, it is unclear if the targeted immune pathways could be adequately stimulated to produce the expected boost in immunity. Likewise, immune-based therapeutics are seldom applied to models of infection where the laboratory animals are treated with optimal regimens of antibiotics as would be expected in patient care. Short of testing the therapeutics under these settings, the role of these conceptually attractive therapeutics would not be amenable to adequate evaluation and interpretation. In summary, highly innovative research has introduced strategies that tilt the host-*S. aureus* balance in favor of pathogen killing. Going forward, these therapeutics should be carefully scrutinized for treatment of staphylococcal conditions where clinical treatment is still suboptimal.

## Data Availability

Not applicable.
